# A mixed methods analysis of U.S. perinatal providers’ attitudes towards culturally relevant infant mental health integration in obstetrical care

**DOI:** 10.3389/fpsyt.2025.1644836

**Published:** 2025-11-14

**Authors:** Akshaya P. Kannikeswaran, Angela Marie Johnson, Meriam Issa, Maria Muzik

**Affiliations:** 1 University of Michigan College of Literature, Science, and the Arts, University of Michigan, Ann Arbor, MI, United States; 2 Program for Multicultural Health, Department Community Health Services, Ann Arbor, MI, United States; 3 Department of Psychiatry, Michigan Medicine-University of Michigan, Ann Arbor, MI, United States; 4 Department of Obstetrics & Gynecology, Michigan Medicine-University of Michigan, Ann Arbor, MI, United States

**Keywords:** pregnancy, mental health, social stigma, psychiatry, primary health care

## Abstract

**Introduction:**

Many women in the perinatal period present to their women’s health providers with mental health concerns, which may impact the well-being of both the mother and baby. In this study, behavioral health consultants (BHCs) specially trained in the Infant Mental Health (IMH) approach, hereafter referred to as IMH-BHCs, were integrated into prenatal clinics whose patient populations include a significant proportion of Black, Indigenous, and People of Color (BIPOC) women. BIPOC women experience unique challenges in receiving culturally responsive perinatal and general healthcare. The aim of this study was to elucidate healthcare providers’ perceptions of IMH-BHC clinic integration on patient and provider outcomes, assess barriers to integrating behavioral health care into obstetric care environments, and understand the impact on quality of care delivery resulting from adding behavioral health support to perinatal populations.

**Methods:**

This was a mixed-methods study conducted in Midwestern United States (Michigan); we collected survey responses from healthcare providers (n=52) on model knowledge and satisfaction; we also conducted a qualitative analysis of in-depth interviews with a subset (n=9). One-on-one interviews were guided by an ethnographic approach and focused on gathering thoughts, attitudes, and perceptions of health providers on integrating IMH-BHCs into their practice. Analysis included individual review, open coding, and thematic analysis of transcribed interviews using a grounded theory approach.

**Results:**

Quantitative survey results reflected high knowledge about and engagement with the model. Survey respondents also endorsed the presence of the IMH-BHC improving scope focus and time management. Two main themes emerged with five sub-themes from our qualitative interviews. The main themes were: 1) initiation and engagement with the IMH-BHC model, and 2) evaluated effectiveness of the IMH-BHC. The sub-themes were: 1) IMH-BHC strategies to engage patients, 2) barriers to care facilitation, 3) continuity of care with IMH-BHC, 4) presence of the IMH-BHC may help aid in timely care, and 5) potential for reduction in provider workload and stress.

**Conclusions:**

Overall, the integration of IMH-BHCs into clinical settings was regarded as beneficial due to decreasing provider workload, increasing accessibility to mental health services for socially marginalized populations, and enhancing patient engagement. Further research is needed to mitigate barriers to IMH-BHC integration.

## 1 Introduction

Reproductive health providers serve as the primary or sole source of healthcare for a substantial proportion of women (1), giving them a central role in addressing both physical and mental health needs. As nearly 70% of all primary care visits are directly or indirectly related to psychiatric problems (2), it is common for women to present behavioral health concerns to their obstetricians/gynecologists (3), especially during the perinatal period. The perinatal period, from pregnancy through the child’s first year, is a time marked by rapid change and increased vulnerability to mental health concerns (4).

Prior research has shown that 8.5-11% of women experience depression during pregnancy and 6.5-12.9% of women experience depression during the first postpartum year (5). Rates of postpartum depression are significantly higher among individuals identifying as American Indian/Alaskan Native (22%), Asian or Pacific Islander (19%), or Black (18%) compared to those identifying as White (11%) (6). Moreover, Black Indigenous and People of Color (BIPOC) women commonly report experiencing a lack of communication, racial discrimination, and bias from providers in perinatal healthcare settings, resulting in patient distrust and underutilization of critical care (7, 8). Beyond prevalence, mental health problems in pregnancy are associated with a greater likelihood of poor pregnancy outcomes, such as preeclampsia, gestational diabetes, preterm birth, etc. (9).

Moreover, perinatal mental illness effects extend beyond the mother to potentially adversely impact the child as well (10). Fetal exposure to untreated maternal mental illness in pregnancy has been linked to difficult infant temperament, impairment in brain development, and subsequent proneness to child mental health problems such as Attention-Deficit/Hyperactivity Disorder (10, 11). Postpartum, maternal mental illness can interfere with responsive mother-infant interactions, impair maternal bonding, and lead to heightened risk for infants to develop insecure attachments and subsequent psychopathology, such as pediatric anxiety and depression (10). Thus, any responsive obstetrical care in the prenatal and/or postpartum periods must not only attend to the mother’s mental health alone, but equally attend to the child’s health, the emerging mother-infant bond, and the relational health between mother and child.

Over the past decade, integration of mental health care into obstetric practices has been rapidly increasing with evidence for positive effects (12, 13). These models in obstetrics were developed as a response to low rates of routine mental health screening and treatment in routine obstetric care. A systematic review of integrated care interventions for perinatal depression in obstetric settings found that these models are feasible, effective, and acceptable to patients and providers, especially when integration is facilitated by leadership support and clear referral pathways. However, the sustainability of practice was challenged by resistance to changing established workflows, time constraints, and a lack of mental health expertise among clinic staff. A randomized control trial comparing integrated geriatric mental health services to enhanced specialty clinic referral similarly showed significantly higher treatment engagement and more patient visits in integrated models, though staff faced challenges adapting to new workflows and complex integration processes (14). Qualitative evaluation of a compassionate care intervention further reinforced staff support for integrated approaches and reported improvements in well-being and patient care, yet sustainability was limited by organizational culture and competing priorities (15). Across these studies, staff training was regarded as essential but variably implemented with training modalities ranging from didactic education, protocolized screening and referral, and interdisciplinary collaboration (15, 16).

Such models of mental health-obstetrics care integration improved perinatal depression outcomes (16) and made behavioral health accessible to populations whose concerns went otherwise undetected or underserved in prior traditional care (14, 15). Especially for vulnerable populations, such as BIPOC women, an integrated mental health care model allows efficient, effective, and accessible mental health care within routine women’s health care. While integrated behavioral health models have demonstrated improvements in screening, engagement, and patient outcomes, there is a persistent gap in equitable access for BIPOC women. Implementation challenges of staff training, workflow integration, and organizational support are magnified in settings serving BIPOC women, who may face additional stigma, distrust, and systemic obstacles. Given these limitations, there is a critical need for evaluation of integrated behavioral health care models that are positioned to address the unique needs of BIPOC women in prenatal care settings as well as the needs of other perinatal patients (8).

Given the necessity to consider both mothers’ and infants’ wellness concurrently, a comprehensive approach is needed that simultaneously addresses the well-being of the mother, her infant’s well-being, and addresses the emerging mother-infant bond (17), particularly in medical settings that serve BIPOC perinatal populations and in ways that are culturally responsive. The Infant Mental Health approach (IMH) (18) has a special focus on the developing dyadic relationship because the health of mother and child is intricately interconnected. Originally, IMH was developed as a home visiting model (18, 19) delivering multimodal, needs-based interventions such as connection to resources, attention to basic needs, emotional support, developmental guidance, and relational psychotherapy to promote reflective capacity and parenting skills. In recent years, principles of the IMH model have been increasingly integrated into prenatal medical care (10, 13, 20, 21). IMH clinicians’ specialized training and knowledge of Social Determinants of Health, trauma-informed care, and cultural humility allow for a relational and multigenerational perspective in the obstetric setting (22), foster maternal and child wellness, inherently address issues of inequity, and ensure care that is inclusive, effective, and accessible (23).

The aim of this manuscript was to extend the current literature on perinatal providers’ perspectives when integrating a culturally responsive and IMH-informed mental health approach into obstetrical care. Piggybacking on a study evaluating such IMH-informed integration of mental health into prenatal medical care, we focus in this paper on staff experience and feedback about such an integration. Data obtained from this study are highly relevant as staff perception has been shown to be a barrier or facilitator of model uptake and implementation (14, 15). A total of ten obstetric clinics in Metro Detroit (Michigan, United States) engaged in a large pragmatic evaluative study in which 7 clinics agreed to integration, that is, an IMH-trained Behavioral Health Consultant (IMH-BHC) was embedded into their prenatal praxis and offered assessments, brief therapeutic interventions, and referrals for other social services, including IMH home visiting to the patients on site. The remaining 3 clinics continued standard care without integration. Whereas integrated mental health as a care model carries tremendous advantage for improved healthcare delivery (13, 20, 21) and patient experience (10), successful implementation requires adaptations to routine clinical practice that may seem burdensome to clinic staff including reshaping practice patterns, modifying established clinical flow, inclusion of behavioral health providers into the medical team, and valuing mental health as a core target for care within team discussions and documentation (24). In this paper, we present the results of a mixed-methods evaluative study of provider perspectives on integrating IMH-BHCs into obstetrical clinics across the Metro Detroit area of Michigan, primarily serving BIPOC women. This study highlights providers’ perspectives on and experience with the IMH-BHC model and discusses the implications of the integration in terms of impact on provider workload and patient engagement. These results will inform the development of culturally responsive IMH and perinatal care interventions and potentially guide other efforts to integrate such models into standard obstetric medical care.

## 2 Methods

### 2.1 Design of parent study

The original pragmatic trial aiming to evaluate whether the integration of the IMH-BHC into obstetrical practice versus delivery of standard care may beneficially impact patient mental health, pregnancy outcomes, and access to needed resources was conducted between 2018 and 2022 and through a community (Starfish Family Services, SFS) - university (University of Michigan, Zero To Thrive) partnership. SFS is a nonprofit human service agency headquartered in Inkster, Michigan, that provides educational and behavioral health services to families and children across metro Detroit. SFS invested in the development and piloting of the IMH-BHC model and partnered with local obstetric clinics across Metro Detroit to implement the model. SFS was highly invested in comprehensive staff training within the obstetric clinics that integrated the model. Each clinic’s training was comprised of an “all-staff” workshop (~4 hours) with targeted follow-up implementation coaching and technical assistance. The training had special emphasis on team‐based care for service provision and on infant mental health/relational care principles, as this was deemed particularly important to assure that the IMH‐BHC (who was a clinician provided by SFS) is truly integrated in the obstetrical team, and that the IMH therapy approach is understood and valued by the obstetrical team members in order to guarantee a coherent messaging to the patients.

A total of ten obstetrical clinics in the Metro Detroit area serving predominantly BIPOC and under‐resourced women participated in the pragmatic trial (10). A total of 158 pregnant women were recruited through this trial prior to 28 weeks’ gestation. Seven clinics adopted (or had already adopted prior to trial startup) the integrated IMH-BHC model (“intervention group”, n=90 women (57%)), while three clinics continued with standard care without integration as a comparison (treatment-as-usual) group (n=68 women (43%)). This selection for integration was self-guided by clinic management. All enrolled women provided data on physical and emotional functioning and risk and resilience twice during pregnancy (at intake ≤28 weeks and between 28–42 weeks of gestation) and three times in postpartum (at 6 weeks, 6 months, and 12 months postpartum). Data collection was conducted by the university partner (University of Michigan, Zero To Thrive). Findings from this pragmatic trial are currently under review.

### 2.2 Design for current sub-study

A sub-study on provider experiences with the IMH-BHC integration was conducted in 2022. A mixed-methods approach was used to study providers’ perspectives on IHM integration to understand, from quantitative survey measures, what is happening as well as why it is happening through qualitative interviews. We sought to understand patterns and trends in IMH-BHC integration and the motivations, frustrations, and modifiable factors behind the patterns. In this way, we hope to detail useful converging insights or discrepancies that invoke fresh perspectives and new, more illuminating explanations for improved interventions (25). The sub-study included both a written online survey emailed to providers and a subsequent virtual interview of providers.

Of the initial ten clinic sites from the parent study, seven clinic sites (six clinics with IMH-BHC integration, and one clinic with standard care) participated in this substudy; three clinics were excluded due to regulatory barriers. We approached clinic managers at each of the seven clinic sites, asking them to email a confidential survey to their respective clinic staff. At the end of the emailed survey, participants could self-select to also provide their full name and email contact if they were agreeable to participate in a follow-up qualitative interview. Participants who self-selected for the interview gave written informed consent prior to the interviews. The study was approved by the University of Michigan Institutional Review Board.

### 2.3 Provider surveys: participants, data collection, and rating

The confidential online survey was emailed to 85 clinic providers or staff, and 52 (61%) providers returned the survey. As the survey distribution was done by the clinic manager, information about reasons for declining and characteristics for self-selection is missing. The survey, tapping into awareness of and satisfaction with the integration model, contained 13 questions; 4 questions asked information about professional role and tenure at the clinic, credentials, and age; 8 questions asked about knowledge and satisfaction with the integrated IMH model (**Table 1**); and the last question asked about willingness to participate in an in-depth qualitative interview on the subject. The survey instrument was developed collaboratively by the research team with input from the clinic manager and IMH-BHCs. Respondents rated their knowledge and satisfaction with the integrated IMH model on a 7-point Likert scale (strongly disagree, somewhat disagree, disagree, neutral, somewhat agree, agree, and strongly agree). To facilitate analysis and consistent with recommendations from existing literature (26, 27), we collapsed responses into three categories: agree, neutral, and disagree. This decision was made due to observed low response frequencies in some categories (somewhat and strongly) and to improve interpretability and statistical power. Participation in the survey was not reimbursed.

**Table 1:** Staff and provider agreement with program satisfaction statements from survey data (n=52).

Statement	Agree	Neutral	Disagree
I feel well-informed about the IMH-BHC model.	**76.9%** (n=40)	**3.9%** (n=2)	**19.2%** (n=10)
Our OB/GYN practice is fully integrated.	**80.8%** (n=42)	**15.4%** (n=8)	**3.8%** (n=2)
I understand the role of IMH-BHCs.	**90.2%** (n=46)	**2.0%** (n=1)	**7.8%** (n=4)
The presence of the IMH-BHCs in our OB/GYN practice saves me time.	**78.0%** (n=39)	**16.0%** (n=8)	**6.0%** (n=3)
Having an IMH-BHC embedded in the practice allows me to better focus on the job I am trained for.	**86.3%** (n=44)	**11.7%** (n=6)	**2.0%** (n=1)
The IMH-BHC in our OB/GYN practice is readily available for consultation and referrals.	**92.0%** (n=46)	**6.0%** (n=3)	**2.0%** (n=1)
I know when to involve IMH-BHCs.	**81.3%** (n=39)	**10.4%** (n=5)	**8.3%** (n=4)
Having an IMH-BHC embedded in our OB/GYN practice has improved patient compliance.	**69.4%** (n=34)	**24.5%** (n=12)	**6.1%** (n=3)

Bolded values for better visibility.

### 2.4 Provider interviews: participants, data collection, and coding

Nine providers self-selected for an in-depth qualitative interview, and eight worked in clinics with integration. The demographics of the interviewees were as follows: average age was 44 years; seven were women, two were men; five self-identified as White/Caucasian, one as Black/African American, and three declined to answer. The majority of staff (n=6) had been at the respective clinic site for at least six years or longer. Three interviewees were physicians, one was a nurse, two were medical assistants, two were front desk staff, and one was an office administrator. The purpose of the interview was to obtain a deeper dive into providers’ perceptions, thoughts, and experiences in daily practice with the integration or lack thereof (see **Appendix 1** for interview guide) and to expound on perspectives shared in the survey in order to learn modifiable factors for enhancing integrated perinatal IMH care. The semi-structured interview was brief (~ 20 minutes) and conducted by phone (n=6) or in person (n=3) in the workplace, with most interviews conducted by phone due to lingering impacts of the COVID-19 pandemic. The first five minutes of the interview were spent asking about the interviewee’s demographics, professional role, clinic tenure, and familiarity with the integrated IMH model. Participants who completed the interview were mailed a $10 gift card as a thank you for their participation.

Interviews were audio-recorded, professionally transcribed verbatim, anonymized, and then thematically coded for salient codes and themes. All interviews were read, coded, and discussed by the first and second authors, both of whom identify as women of color with no pre-existing relationship to the participants. The first author is a university student training in gender and health; the second author is a PhD sociologist with an extensive qualitative research background. Each researcher individually coded the transcripts into salient themes, which was followed by constant comparative analysis until all transcripts were analyzed in agreement to ensure consistency. Salient themes were decided based on codes’ frequency, extensiveness, intensity, specificity, and perceived importance by participants. Interpretation of results was analyzed using grounded theory analysis (28). Results were guided by the Standards for Reporting Qualitative Research (SRQR). The SRQR reporting checklist is provided in Supporting Information: **Appendix S1**.

## 3 Results

### 3.1 Survey data

Demographics of providers (n=52) who filled out survey data were as follows: the majority of the providers (n=36) had been at the clinic site for less than ten years. 16 respondents were physicians, 8 were nurses, 19 were medical assistants, and the remainder were front desk staff or office administrators. **Table 1** presents the response frequencies to the eight knowledge/satisfaction survey questions across the 3 response categories, with some missing data due to some respondents declining to answer questions. Overall, knowledge about and satisfaction with the model were high. More than 75% of respondents felt well informed about the model (9/16 front desk staff, 14/19 medical assistants, 8/8 nurses and 11/16 physicians), felt their clinic was practicing an integrated model (8/9 front desk staff, 16/19 medical assistants, 8/8 nurses and 10/16 physicians), and voiced understanding of when and how to involve the BHC (7/8 front desk staff, 14/18 medical assistants, 8/8 nurses and 11/15 physicians). Moreover, respondents rated the benefits of an IMH-BHC integrated into their obstetrical clinic as very positive and stated that having quick and easy access to the BHC and their services to the patients allowed the obstetrical provider to “better focus on the work [they] were trained to do” (7/8 front desk staff, 16/19 medical assistants, 8/8 nurses, and 13/16 physicians) and saved the provider time (7/8 front desk staff, 13/18 medical assistants, 7/8 nurses, and 12/16 physicians).

### 3.2 Interview data

Demographics of providers (n=9) who provided insight were as follows: average age was 44 years; seven were women and two were men; five self-identified as White/Caucasian, one as Black/African American, and three declined to answer. The majority of providers (n=6) had been at the clinic site for at least six years or longer. Three respondents were physicians, one was a nurse, two were medical assistants, and the remainder were front desk staff or office administrators. Feedback from providers shared during the interviews parallels and consistently reflects insight shared in the surveys. Providers feel that the IMH-BHC model represents a positive contribution to obstetric care settings by providing a comprehensive, one-stop shop that offers critical physical and mental health support for patients. They acknowledge that the model helps counter systemic factors, distrust, and social life demands that have historically prohibited and inhibited access to adequate care. Additionally, ideas surfaced during interviews reveal potential strategies for enhancing care, particularly for BIPOC patients. The following details two major themes and five separate sub-themes that emerged from thematic analysis of interviews.

#### 3.2.1 Theme 1: Initiation and engagement with the IMH-BHC model

##### 3.2.1.1 IMH-BHC strategies to engage patients

Interviewed participants discussed various considerations on how to best introduce the model. For example, some cited specific circumstances, such as a patient scoring high on a psychosocial or mental health risk screener, as a time when they would refer to the integrated IMH-BHC.

*“So, when we’re screening our newly pregnant moms, we go through a screener, and so if they are positive on that, then we offer services. I also like to throw in for any patients who have maybe a history of anxiety or depression.”* - Female nurse at integrated site

*“I just refer them if they have an issue, like high GAD or PHQ score, or if they have social stressors that are going on that I hear about any kind of hard social situation.”* - Female doctor at integrated site

Providers commonly noted the importance of avoiding use of the term “social worker” because of its historically negative meaning based on perceptions and experiences with patients, families, community members, and others, particularly within communities of color. Providers instead chose to describe the IMH-BHC as someone who could offer a “helping hand” to patients struggling with mental health or personal life challenges. While this framing reflects efforts to reduce mistrust in patient interactions and increase utilization of care, it also highlights how deeply entrenched perceptions and systemic stigma of social work and mental health services are for socially marginalized populations. As was similarly shared in the surveys, providers’ insight reflects their interest in repairing patient-provider trust and bolstering the chances that a patient will engage in care and exercise compliance in continuity of care.

*“You know, one of the things I never say is, I don’t say anything related to social work. Now, what I don’t know, I don’t know if that turns patients on or off, but I think sometimes there is a huge trust issue with some of our patients, and that’s because some of them have been in the system in the past or they’ve had some previous experiences. So I put more of a gentler spin on it by saying they’re here to help you cope with whatever life stressors.”* - Female nurse at integrated site

##### 3.2.1.2 Barriers to care facilitation

Survey respondents discussed that IMH-BHC integration enhances the overall perinatal care experience. Feedback from those interviewed reiterates this and also illuminates some barriers to successful care. Specifically, several providers noted logistical barriers in connecting patients to IMH-BHC care in real time. Despite being embedded in the clinic, the IMH-BHC was not always available when needed (e.g., talking to another patient in another room, or absent from the clinic on that day for vacation or illness). The balance between demands for and availability of care decides program success.

*“There have been a few times where we’ve had a situation from the nursing standpoint we do see a lot of like the anxiety/depression issues more so than any of the other like housing or food issues, but where she’s not here, and so that kind of puts us where we want to like close the gap, get the patient what they need, but then, you know, we have to wait a couple days.”* - Female nurse at integrated site

Although these barriers are logistical in nature, they demonstrate how integration, although important and needed, remains tenuous without organizational structures that support and provide an adequate number of BHCs that can offer consistent access to patients. Especially in contexts where patient trust in the healthcare system is limited, even small disruptions can reinforce negative perceptions about mental healthcare.

The quality of BHC clinical and communication skills and practice was also highlighted as important. Proactive approaches to patient care by IMH-BHCs (e.g., frequent checking in on patients, introducing themselves to patients, etc.) facilitated better patient response and engagement. This expands on the positive insight from the written survey, that the ability of the BHC to build trust and rapport through proactive engagement is an essential component in program design and success.

*“So we have had two different [BHC] therapists here. [Previous BHC] was a little more assertive, maybe. She would go and introduce herself to everybody. Yeah, and I guess if patients are reluctant anyway, if they’re not approached, that might be one more barrier.”* - Female nurse at integrated site

In sum, providers (via both survey and interviews) expressed appreciation for the value and impact of integrated IMH-BHCs. Nevertheless, some patients declined to utilize available IMH-BHCs. According to providers, a number of patients were reluctant or expressed concern about having a “stranger” call them or visit in their home, perhaps signaling patients’ mistrust of healthcare providers and healthcare systems.

*“We get kind of a mix, we get some patients who are all about it and wanting the resources, and others that are kind of more hesitant, they still will talk with them and then they kind of determine at that point, like, it’s not something that they’re wanting to move forward with, but I think we do get a fair share of patients who do utilize those services, which is great because I know they’re appreciative of it.”* - Female nurse at integrated site

*“[Provider] and I have discussed that, that we do think [BHC] is a good asset to the clinic, but again, it’s the patients. They just don’t want to be bothered. They don’t want her calling. They don’t want her coming to the home.”* - Female medical assistant at integrated site

*“They don’t want a stranger in their home. They don’t know if their partner or their husband will like it.”*

- Female medical assistant at integrated site

More work is needed to illuminate factors that influence marginalized patients’ willingness to utilize IMH-BHCs. Nevertheless, it is clear that providers consider incorporating IMH-BHC programs as worthwhile and vital.

##### 3.2.1.3 Continuity of care with IMH-BHC

Our interviewee feedback indicates that patients and providers feel there are tremendous benefits to successful continuity of care between IMH-BHCs and patients, but acknowledge variation in patient utilization over time, with some providers describing a “mixed bag” of engagement. Three providers shared that patients consistently saw the IMH-BHC and continued to follow up with appointments long-term, but underscored that real-time access to the IMH-BHC was critical for success as patients were less likely to accept care at a later time point.

*“I think maybe less than. Maybe 2% [of patients] don’t take advantage of it….they almost always do, I would say 98% of the time, [patients] say, sure, I’d be glad to talk to her [BHC].”* - Male doctor at integrated site

*“I had a young mom, I think she stated to me that she was a little bit overwhelmed, and then I said, can I give [BHC] your number? She can help you with some things. She allowed [BHC] to call her, and then she did talk to [BHC], but then she told [BHC] she didn’t need her services.”* - Female medical assistant at integrated site

This finding points to the importance of immediacy in integrated models. Engagement was strongest when the IMH-BHC was introduced in real time within the context of a trusted clinical team environment.

#### 3.2.2 Theme 2: Evaluated effectiveness of the IMH-BHC

##### 3.2.2.1 Presence of the IMH-BHC may help aid in timely care

Interview discussion with providers echoes the insight we learned from providers in written surveys. Despite numerous barriers, providers find it highly advantageous to have access to a BHC in the clinic because it permits targeted, impactful healthcare, saves time, and improves the chances that patients will utilize care and get the healthcare they need.

Providers consistently described that IMH-BHCs facilitate timely access to critical mental health resources. At integrated sites, the presence of the BHC allowed providers to address mental health concerns promptly and effectively, often immediately following screening results. This integration appeared to bridge gaps that previously existed when providers lacked the resources or personnel to respond to patients’ social and emotional needs. By delegating these conversations to a trained specialist, providers could ensure that patients were connected to appropriate care while focusing on their clinical responsibilities.

*“They’ll score high on their postpartum, and sometimes it’s nice just to have [BHC] there. I know we can’t have her at all times, but it is nice because like, when they score high on the postpartum, I’ll give it to [BHC], like because she scored high, you might want to introduce yourself.”* - Female medical assistant at integrated site

*“Patients who have known pre-existing anxiety or depression, a new diagnosis of postpartum depression, I would send those to [BHC] because I’m going to probably start them on meds and talk to them about the importance of talk therapy and have [BHC] help them get connected and continue to check in with them and make sure they have connected with someone … It’s really nice to have some type of resource just because prior to that, you had nothing to offer them.”* - Female doctor at integrated site

*“[referring to a potentially suicidal patient who had called in] She [the BHC] then contacted the patient shortly after I did, so I thought that was really great, because sometimes you don’t always expect to get that, where we previously had tried getting these moms that are having, you know, issues in, and it was like, you know, a month out before they would be able to get seen, and I’m like, no, that’s not helpful!”* - Female nurse at integrated site

These examples illustrate that the IMH-BHC not only helped provide a pathway for care but also offered reassurance to providers that patients’ needs were being met efficiently. In contrast, the interview from the comparison (treatment-as-usual) site indicated reliance on external resources, suggesting that patients’ needs potentially may be less readily addressed at this site without an embedded behavioral health professional. However, given the limited data available from treatment-as-usual sites, these observations are presented cautiously.

*“I think it’s super important to address those needs during pregnancy, … depression/anxiety … in addition to the social influencers of health …, we don’t have case managers in the office; we don’t have that level of hand-holding available that I think would be beneficial to a lot of patients…., we’re very limited in almost what we can offer …. It’s kind of like an internet search engine using data to sync people into resources but it’s very impersonal. If those gaps are kind of outside the scope of just straight nursing, typically the idea would be to try to get them synced into social work or a program that could provide more support…… I still think that there is a lot to be desired in that resource for patients.”* - Female nurse manager at treatment-as-usual site

##### 3.2.2.2 Potential for reduction in provider workload and stress

Interviewed providers also report that the IMH-BHC integration helped reduce workload and stress by managing sensitive conversations that patients might feel uncomfortable having with physicians or nurses. This delegation allowed providers to focus on other clinical responsibilities while ensuring patients received targeted support. Beyond patient care, the BHC also may serve as a support system for staff, illustrating the broader benefit of behavioral health integration within the clinic.

*“But I think more from a bigger perspective, and the doctors may be able to speak better on this, but I think they often are seeing patients who maybe they’re reluctant to mention things to the nursing staff.”* - Female nurse at integrated site

*“Some staff that are maybe having their own issues have gone to him [BHC] and he’s talked to them and given them resources and things, and, obviously, everything’s very confidential, but I think that’s a great thing as well, because being in healthcare, we’re always putting everyone else ahead of us.”* - Female nurse at integrated site

Together, these findings suggest that integration of the IMH-BHC may function to both optimize patient care and mitigate provider stress, enhancing overall clinic efficiency.

## 4 Discussion

This study aimed to understand perinatal health providers’ experiences across clinics in Metro Detroit, in Michigan (United States) in integrating IMH-BHCs into the clinical setting, identify patient access factors, and consider potential strategies for enhancing IMH-BHC integration. This is particularly important for BIPOC patients for whom lingering effects of historic harm and deference in research and healthcare practices manifest as distrust and underutilization of available healthcare. Providers’ written survey and interview results both characterize IMH-BHC integration as a positive and impactful model of care. Interview outcomes provide details of major program benefits. These include improved patient response, eased provider workload, and individualized mental health support delivered with humility and understanding, all of which reinforce previous work, particularly for those patients who present with complex physical health, mental health, and personal life conditions. Consistent with other recent work (29), our interview data showcase providers’ perception of model benefits to continuity of care for patients. The study implications are timely for both providers and the BIPOC women they serve, who experience a higher incidence of adverse physical and mental health conditions, often shaped by social and structural factors (30). Although these populations require care, they may feel discouraged from seeking help for fear of experiencing racial trauma from the very healthcare providers and systems poised to serve them (7, 8).

First, the model, when delivered in a proactive, personalized, and efficient manner, can help ensure that BIPOC patients receive mental health care and attention alongside primary care in a way that is impactful and culturally relevant. Prior research demonstrates that patients are more likely to follow up with behavioral health treatment when services are integrated into the clinic (31, 32). Insight gathered in our study, particularly with regard to patients experiencing postpartum depression or anxiety, reinforces previous work stating that IMH-BHCs satisfy a critical need for timely service and support (29). The integration of perinatal health care and humility in this care is especially recommended for addressing the higher prevalence of perinatal mood disorders, including postpartum depression and anxiety, among Black mothers, many of whom juggle paid work outside the home (33). This is especially crucial given the prevalence of employed pregnant women and mothers with young children in the US (34) who may be less inclined to visit a mental health clinician outside a prenatal visit. Moreover, the integration of behavioral health care into the perinatal clinic allows patients to be seen concurrently in a familiar environment, ensuring convenient, less disjointed, and less stigmatized mental health care (35). Providers further noted how an in-person IMH-BHC led to a swifter and positive connection between the BHC and patient. Early intervention in mental health disorders has been shown to decrease the severity of disease (34) and allow for a more personalized approach to mental health interventions tailored to the social and cultural context of the patient (36).

Comparing these findings to studies conducted in other countries reveals both commonalities and distinctions in the integrational of behavioral health care into perinatal settings. For instance, research from the United Kingdom and Australia has similarly shown that embedding mental health interventions within maternity or primary care clinics improves access and is associated with improved patient satisfaction and increased specialist referrals, particularly among minority populations (37, 38). However, unlike in the United States, these programs often benefit from the universal healthcare systems that facilitate funding and standardization of integrated care models. In contrast, fragmented insurance systems in the United States may hinder consistent implementation. Studies in Canada and Scandinavian countries further emphasize the value of culturally competent perinatal mental health interventions for immigrant and minority women (39, 40), underscoring the global need for responsive integration strategies such as IMH-BHCs. These comparisons emphasize that though the benefits of integrated care are consistent and well-documented, structural differences across healthcare systems can shape the feasibility and sustainability of such models.

Third, the physical presence of an IMH-BHC in a clinic may ease primary care providers’ (PCPs) workload by dedicating focused psychosocial support. A prior study showed that although patient encounters involving BHCs had a longer total duration with an increase of approximately 11.23 minutes, the time primary care providers directly spent with the patient decreased greatly by about 11.67 minutes (41). These results suggest that BHCs might help decrease the time and talent burden placed on PCPs by addressing mental health issues earlier, allowing providers to focus more on medical concerns. Our study outcomes reinforce that the BHC permitted medical providers to focus more on the specific clinical role they were trained in, rather than behavioral health.

Providers also described challenges. For example, real-time unavailability of a BHC posed a major barrier to patient engagement with mental health services. There is a shortage of behavioral health specialists nationally that is only expected to rise throughout the years (42), and at times, there were vacancies despite integration. Burnout caused by chronic workload, lack of control, and lack of support may also lead to higher intent to leave, depersonalization, and poor morale among BHCs (42) that could contribute to the issue of unavailability raised by providers. IMH-BHC, as an individual-level intervention, will be insufficient without also addressing structural issues, such as workload inequities, lack of leadership diversity, and underfunding (43). The Integrated Workforce Trauma and Resilience (IWTR) Model offers a useful framework for understanding and addressing these challenges and considers systemic solutions to mitigate burnout, improve retention, and advance global mental health workforce equity. Additional effort is needed within the IWTR lens to address organizational and systemic factors influencing workforce retention and to pursue strategies that build organizational and provider resilience through policy and education (43). As discussed, BIPOC women may experience increased stigma regarding mental illness and may fear discrimination; however, cultural norms are shifting, indicating increasing numbers of Black/African American women seeking out professional mental healthcare, especially in programs that remove transportation and cost barriers (44). Cultural insensitivity within social services may imbue hesitation, reluctance, anxiety, and ultimately the refusal of BHC support by BIPOC patients, as noted by several providers in this study (45). This further exemplifies the need to increase diversity in healthcare providers, including BHCs, to provide more culturally competent care.

## 5 Limitations

This study has several limitations. First, several providers declined to self-identify race, age, and clinic tenure, which limited our understanding of the cultural context and implications of their responses (46). Secondly, difficulty in securing more interviews from clinicians due to busy clinic environments with inherent time demands resulted in a relatively small sample size of nine interviewees, limiting novel contributions that additional surveyed providers may have provided in interviews. Third, both our survey and interview samples were self-selected, which may introduce bias and can lead to systematic differences between study participants and the target population (47). Fourth, the limited time availability of providers could also lead to difficulty discerning all relevant ideas in provider interviews. Fifth, interviews with providers were also completed virtually, without the advantage of observing non-verbal body language as an important supplement to verbal language and tone (48). Sixth, our interview began with questions regarding demographic information of providers, which can introduce priming effects that potentially influenced later responses to certain interview questions due to altered cognitive frames for the duration of the interview (49).

## 6 Conclusions

Feedback from providers in this study suggests that there is clear value in integrating mental health services into prenatal clinics. They report that IMH-BHC integration encourages timely and culturally aware mental health aid for patients, addresses socio-emotional needs, and provides in-clinic support for staff, potentially improving the efficiency and quality of patient service delivery. Insights shared in interviews also reflect difficulties in patient engagement related to patient distrust, shaped by historical, social, and structural context, and lack of timely access to IMH-BHCs in the clinic. Although IMH-BHC integration offers multiple benefits, program design must incorporate strategies to address cultural concerns. Effective approaches may include establishing permanent behavioral health services within prenatal clinics to ensure consistent availability, focusing more on building rapport and trust, and addressing stigma to facilitate acceptance of mental health support. This emphasizes that successful integration requires not just embedding necessary providers but aligning organizational systems to ensure the immediacy and continuity of follow-up appointments. Additionally, quality of care must reflect humility and cultural literacy. Evidence suggests BIPOC perinatal populations require access to respectful, attentive, and culturally knowledgeable healthcare that acknowledges limitations of disjointed large healthcare systems, but are capable of circumventing and buffering barriers to deliver the best care. Further research should address gaps in culturally responsive care, including the development and validation of adapted screening tools for diverse populations, and evaluate policy-level interventions for this specific demographic. Conclusions are drawn from a limited and self-selected sample of integrated sites and a single treatment-as-usual site; thus, they should be interpreted cautiously. Further research with larger, randomized samples is required to elucidate the most effective strategies for integrated perinatal mental healthcare and to advance health equity.

## Data Availability

The raw data supporting the conclusions of this article will be made available by the authors, without undue reservation.

## Appendix 1 Interview Guide.

*FOR BHC SITES:*

You responded in the survey that you are [familiar…. Not familiar] with the BHC program. How would you describe it to a patient who has never heard of it?

Do you know the name of your clinic’s BHC? Would you know how to reach them if you wanted them to meet with a patient? What would that process look like? Have you personally referred a patient to your clinic’s BHC?

Have you ever heard the term Infant Mental Health? What does that mean to you?

How often is the BHC at your site? (Do you know)? How do you communicate with them? (In person? Phone? Email)?

Do you know if they write notes in your clinic’s EMRs? Do you know where to find them? If so, have they helped you provide better care/understanding?

Have there been times when you would have liked to refer a patient to the BHC but the BHC was not at the clinic? What did you do then? Are there things that would make it easier for you to work in partnership with the BHC?

Can you think of an example where you saw the BHC really help a family out? What did that look like? Can you think of an example where maybe the BHC program wasn’t as helpful as you would have hoped it would be? What do you think went wrong?

What do you think having a BHC adds to the clinic? What do you wish worked better or differently?

*FOR TREATMENT-AS-USUAL SITES:*

We know that a lot of women face multiple challenges during and after pregnancy, everything from lack of resources like diapers or formula, to lack of social support, to unsafe living conditions or violence at home. What role do you think the OB clinic should play in helping families meet their needs? What other resources are you familiar with that sometimes help new parents out? What would the ideal care look like?

What have you personally done to help patients who are having a particularly hard time? What have you seen colleagues do?

What do you think your clinic could do to improve their services for patients?
